# Macular Structure and Microvasculature Changes in AIDS-Related Cytomegalovirus Retinitis Using Optical Coherence Tomography Angiography

**DOI:** 10.3389/fmed.2021.696447

**Published:** 2021-08-13

**Authors:** Kui-Fang Du, Xiao-Jie Huang, Chao Chen, Wen-Jun Kong, Lian-Yong Xie, Wen-Bin Wei

**Affiliations:** ^1^Department of Ophthalmology, Beijing Youan Hospital, Capital Medical University, Beijing, China; ^2^Department of Infectious Diseases, Beijing Youan Hospital, Capital Medical University, Beijing, China; ^3^Beijing Tongren Eye Center, Beijing Key Laboratory of Intraocular Tumor Diagnosis and Treatment, Beijing Ophthalmology and Visual Sciences Key Lab, Beijing Tongren Hospital, Capital Medical University, Beijing, China

**Keywords:** AIDS, cytomegalovirus retinitis, macular, structure, microvasculature, optical coherence tomography angiography

## Abstract

**Background:** Cytomegalovirus retinitis (CMVR) is a crucial blind-causing disease of AIDS-related ocular opportunistic infection. The CMVR lesions produced retinal necrosis. It is not entirely clear whether CMVR eyes without macular-involved necrotic lesions may have subtle macular damage. In this study, we conducted a cross-sectional study using optical coherence tomography angiography (OCTA) to evaluate macular microvasculature and structure in eyes with AIDS-related CMVR.

**Methods:** Acquired immune deficiency syndrome (AIDS)-related CMVR patients (active and inactive CMVR) and healthy controls treated in the Department of Ophthalmology, Beijing Youan Hospital, Capital Medical University between August 25, 2019, and October 18, 2019, were recruited. All OCTA parameters, including the foveal avascular zone (FAZ), retinal vessel density (VD), choroidal vascularity index (CVI), retinal thickness, and choroidal thickness, were compared between groups after the signal strength was corrected.

**Results:** Signal strength in the 3 × 3 and 6 × 6 mm scan patterns was significantly weaker in the inactive CMVR group than in the control group (both *p* < 0.001). After adjusting for signal strength, heterogeneity in the central fovea and parafoveal quadrants was present with a shift toward lower macular chorioretinal vasculature, decreased full choroidal thickness, and thicker retinal thickness in the active and inactive CMVR groups. The retinal nerve fiber layer (RNFL) and inner nuclear layer (INL) were significantly thicker in the active and inactive CMVR groups than in the control group (all *p* < 0.05). For photoreceptor-retinal pigment epithelium (PR-RPE) thickness, no significant differences were found in any quadrant between groups. Foveal avascular zone areas were not significantly different among the three groups (*p* = 0.053).

**Conclusions:** Subtle macular structure and microvasculature damage still existed in CMVR eyes without macular-involved necrotic lesions. The results of our study are helpful for a deep understanding of the damage caused by CMVR.

## Introduction

Cytomegalovirus retinitis (CMVR) is an all-important blind-causing disease but was once neglected in people with acquired immune deficiency syndrome (AIDS) (1). In recent years, apart from diagnosis and treatment, many studies on CMVR have focused on retinal imaging, including fundus photography, fluorescein angiography, fundus autofluorescence, spectral domain-optical coherence tomography (OCT), and adaptive optics (2). However, optical coherence tomography angiography (OCTA) has rarely been mentioned in patients with AIDS. For most CMVR lesions in the peripheral retina, it is not clear whether CMVR eyes without macular-involved necrotic lesions may have subtle macular vascular damage.

Optical coherence tomography angiography in recent years has been a very popular technique in various ocular studies. The unprecedented strength is the near-histology level visualization and quantitative measurement of retinal and choroidal blood density without contrast agent injections (3). The advancement of high-resolution retinal imaging could greatly facilitate the diagnosis and monitoring of chorioretinal disease (4, 5). Optical coherence tomography angiography has been studied in the fields of uveitis, glaucomatous or non-glaucomatous optic neuropathy, choroidal nevi, and systemic disease (6–10). The application of OCT and OCTA in the diagnosis and management of uveitis has also been extensively studied. The OCT/OCTA findings varied according to different types of uveitis and process: increased blood flow in the disc and macular areas during acute stage; epiretinal membrane during chronic stage; cystoid macular edema (CME) along with decreased capillary density (11). For CMVR lesions, OCT images also showed various microstructural abnormalities including vitreous changes, retinal disruptions, hyper-reflective retinal deposits, and retinal vasculature (12). But the application of OCTA is surprisingly limited in AIDS-related CMVR patients.

This study was designed to quantify vessel density (VD), foveal avascular zone (FAZ) area, choroidal vascularity index (CVI), retinal thickness, and choroidal thickness by OCTA in patients with CMVR (active and inactive) and in normal control eyes. Parameters were compared between groups.

## Materials and Methods

### Participants

This prospective observational study was conducted by the Declaration of Helsinki. The ethics committee of Beijing Youan Hospital, Capital Medical University, approved the study (LL-2018-150-K). Both oral and written informed consent were provided to each subject before examinations were performed.

Patients diagnosed with AIDS-related CMVR were recruited between August 25, 2019, and October 18, 2019. Age-matched healthy participants without ocular diseases, previous surgical history, and cardiovascular or cerebrovascular diseases were recruited as controls.

All subjects underwent routine ocular examination, including slit-lamp biomicroscopy, axial length, ultrawide-field (UWF) fundus imaging, and dilated fundus examination with indirect ophthalmoscopy. For patients with AIDS, information regarding medical history, duration of HIV, treatment with combined antiretroviral therapy (cART), and CD4 nadir counts was collected. Medical histories, such as diabetes and hypertension, were collected.

Cytomegalovirus retinitis was clinically diagnosed by an experienced retina specialist with indirect ophthalmoscopy (13). The main retinal manifestations of active CMVR are brush-fire or haemorrhagic necrosis lesions, opaque granular lesions, and frosted branch angiitis (2, 14). As the lesions progressed, CMVR was divided into two subgroups, patients with active or inactive CMVR lesions (15), by experienced retina specialists according to the opacity of CMVR lesion borders: active CMVR lesions represented obvious opacity (mild, moderate, severe, very severe). Lesions showing inactive scars were defined as inactive CMVR lesions.

The classification of CMVR location was based on zones: zone 1 is confined to two papillary diameters from the fovea and one papillary diameter from the edge of the optic nerve; zone 2 refers to areas between the border of zone 1 and the equator; and zone 3 refers to the rest of the peripheral retina (16). We focused on the macular changes of CMVR eyes without macular-involved necrotic lesions. Only CMVR lesions in zone 2 and zone 3 were away from the macular area and included in the study, while CMVR lesions in zone 1 were excluded because of the macular involvement.

### Image Acquisition and Processing

Optical coherence tomography angiography examinations were performed with a commercial swept-source (SS) OCT device (VG200; SVision Imaging, Ltd., Luoyang, China) as described previously (17). This commercial device containing contained an SS laser with a central wavelength of around approximately 1,050 nm (990–1,100 nm full width) and a scanning rate of 200,000 A-scans per second. Two different scan patterns centering on the fovea were conducted in this study: areas of 3 ×3 and 6 × 6 mm. The non-flow area of the FAZ was measured in the 3 × 3 mm scan pattern. The VD, CVI, and average thickness were calculated in the inner circle of the Early Treatment of Diabetic Retinopathy Study (ETDRS) chart of the 6 × 6 mm scan pattern: a 1-mm diameter ring was centered on the fovea; the parafoveal area was confined to the area from the inner ring to the outer 3-mm diameter ring; and radial lines defined parafoveal sectors (superior, inferior, nasal, and temporal).

The retinal VD in the superficial vascular plexus (SVP) and inner vascular plexus (IVP) was analyzed. The SVP located between the inner limiting membrane (ILM), and the junction between the inner plexiform layer and the inner nuclear layer (IPL-INL). The IVP located between the ILM and the junction between the inner nuclear layer and the outer plexiform layer (INL-OPL).

The CVI is a three-dimensional index that refers to the ratio of the choroidal vascular luminal volume to the total choroidal volume and represents the volumetric choroidal vascular density. Macular structural changes were calculated using thickness measurements, including the full retinal thickness, retinal nerve fiber layer-ganglion cell layer-inner plexiform layer (RNFL-GCL-IPL) thickness, RNFL thickness, GCL-IPL thickness, inner nuclear layer (INL) thickness, photoreceptor-retinal pigment epithelium (PR-RPE) thickness, and full choroidal thickness with the 6 × 6 mm scan pattern.

The software removes artifacts and retains the original real blood vessels with the SS-PAR algorithm. For cases with segmentation errors, the manual corrections can fix it and can be further evaluated by a third expert. The image quality was automatically calculated using signal strength by the OCTA device. Eyes with other ocular diseases or failure to cooperate with the OCTA exam were excluded from this study.

### Statistical Analysis

The statistical analysis was performed with SPSS 25.0 software. Independent-samples *t*-tests were used to compare continuous data between groups. The chi-square test was used to analyse the distribution of gender. For normal control individuals, data from both eyes were selected. Taking the signal strength as the covariance, differences in all OCTA parameters (FAZ, VFD, CVI, and average thickness) between eyes with active CMVR, eyes with inactive CMVR, and normal eyes were tested by one-way ANOVA and Bonferroni tests. A value of *p* < 0.05 was accepted as statistically significant.

## Results

### Demographic Data

Based on clinical examination, 34 eyes from 23 patients were diagnosed with CMVR. Four eyes were excluded because of the destruction of the macular area, which made it impossible to locate the macular fovea. Six eyes were excluded because of severe vitreous opacity, which made it impossible to capture the OCTA image. Finally, 24 eyes from 17 patients with CMVR lesions in zone 2 and zone 3 were eligible for this study. The mean CD4 count for patients with AIDS was 128.65 ± 104.56 cells/μl. The median axial length for CMVR eyes was 24.25 mm (interquartile range (IQR), 23.50–25.49 mm). The witting duration of HIV infection was 12 months (IQR, 4–42 months). All patients were free of the coexistence of diabetes or hypertension and were undergoing cART at the inception of the study. In addition, the study recruited 32 eyes from 16 healthy persons without known ocular disease. There was no significant difference in age, sex, or axial length distribution between the CMVR group and the healthy control group (*p* = 0.444, 0.576, and 0.656, respectively) (Table 1).

**Table 1:** Demographic characteristics of patients included in the study.

**Characteristic**	**Patients**	**Controls**	***p-*value**
Age (years), mean ±*SD*	39.71 ± 9.66	37.38 ± 7.40	0.444
Gender (no. male:no. female)	15: 2	13: 3	0.576
Axial length (mm), median (IQR)	24.25 (23.50–25.49)	24.06 (23.53–25.03)	0.656
CD4 nadir count (μl), mean ± *SD*	128.65 ± 104.56	–	–
Duration of HIV (mos), median (IQR)	12 (4–42)	–	–

*HIV, human immunodeficiency virus; SD, standard deviation; IQR, interquartile range*.

### En Face OCTA Features

**Figure 1:**
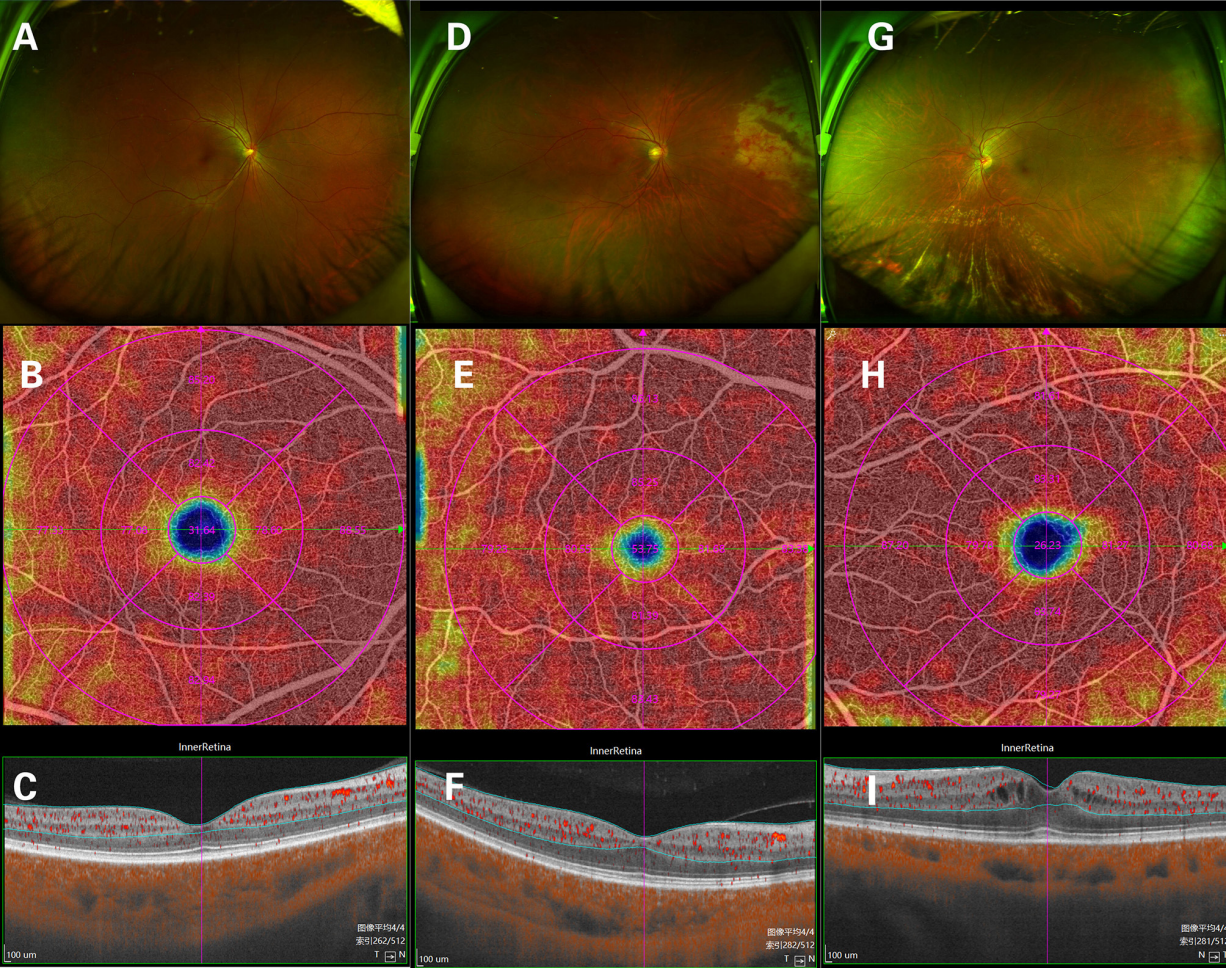
The en face OCTA images of the inner retinal vessel density (VD) and corresponding B-scans (6 × 6 mm scan pattern) in a normal eye **(A–C)**, an active CMVR eye **(D–F)**, and an inactive CMVR eye **(G-I)**.

**Figure 2:**
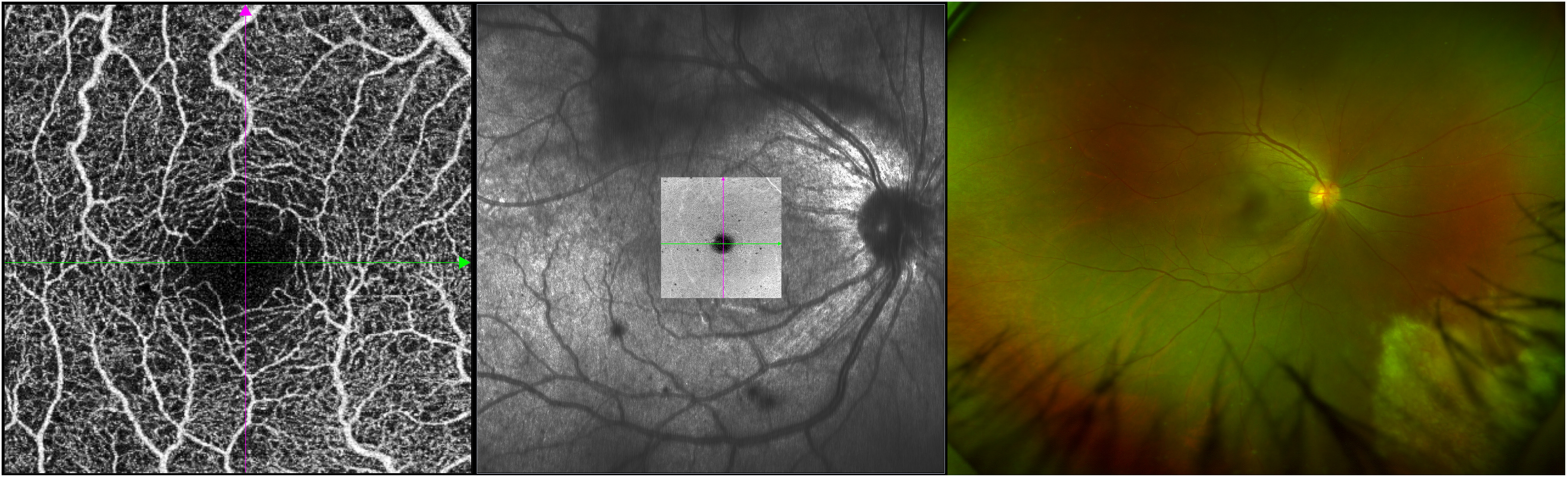
An example of active CMVR in the right eye showing vitreous opacity in the OCTA image (left), en face image (middle), and ultrawide-field (UWF) fundus photograph (right).

**Figure 3:**
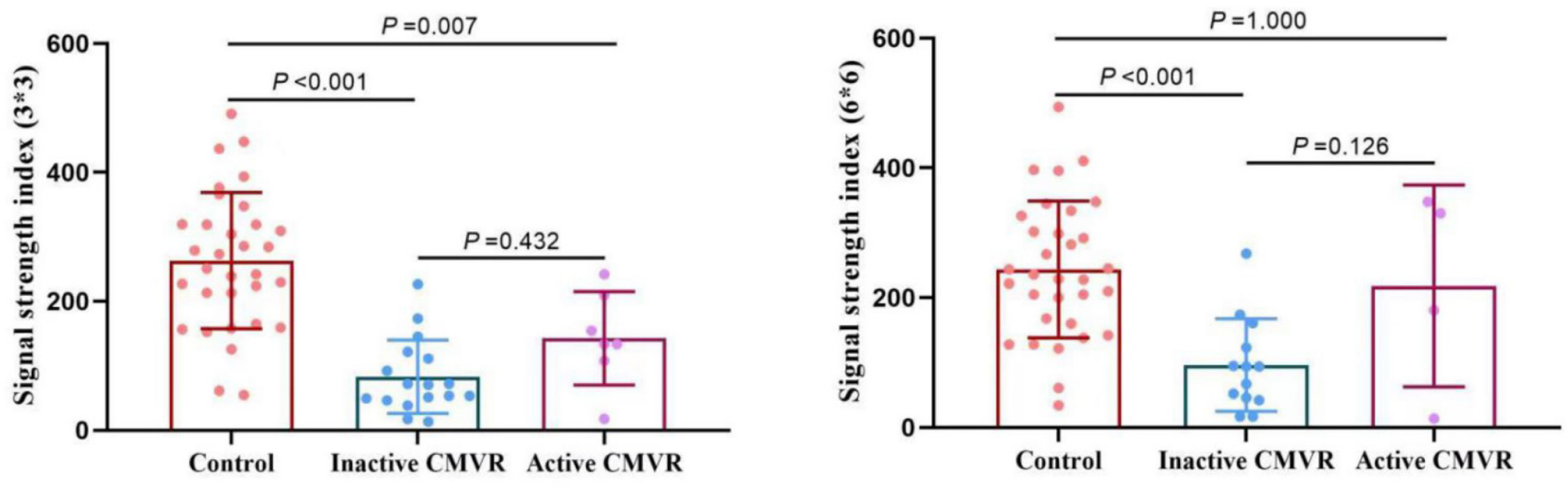
Comparison of the signal strength in the 3 × 3 mm (left) and 6 × 6 mm (right) scan patterns between groups.

Clinically, 24 eyes were diagnosed with CMVR: 7 eyes with active CMVR and 17 eyes with inactive CMVR. The en face OCTA images of the inner retinal VD from different groups and corresponding B-scans (6 × 6 mm scan pattern) with OCTA signals are presented in Figure 1. The en face OCTA images of 16 eyes revealed vitreous opacity (Figure 2): 14 eyes had inactive CMVR (14/17, 82.4%), and two eyes had active CMVR (2/7, 28.6%). Four eyes revealed cystoid macular oedema (CME): three eyes with inactive CMVR (3/17, 17.6%) and one eye with active CMVR (1/7, 14.3%), which were consistent with characteristic features on OCT B-scans. Compared with the control group, the signal strength in both scan patterns was significantly decreased in the inactive CMVR group (both *p* < 0.001); the signal strength in the active CMVR group was significantly decreased in the 3 × 3 mm scan pattern (*p* = 0.007) but not in the 6 × 6 mm scan pattern (*p* = 1.000) (Figure 3).

### Macular Microvasculatural Changes

Supplementary Table 1 shows all the microvasculature parameters in both the central fovea and parafoveal areas (superior, inferior, nasal, and temporal) in the three groups. After adjusting for signal strength, all parameters were compared between groups with Bonferroni correction. The difference in FAZ areas in the three groups was not significant (*p* = 0.053). The macular microvasculature damage was subtle and showed heterogeneity with a shift toward lower superficial and inner retinal VD in the active and inactive CMVR groups. Except for the temporal CVI, the CVI was significantly reduced in the central fovea and superior, inferior, and nasal parafoveal quadrants in the inactive CMVR group (all *p* < 0.05) but not in the active CMVR group (Table 2).

**Table 2:** Statistical results of OCTA parameters of macular microvasculature for all groups.

	**Central Fovea**		**Superior**		**Inferior**		**Nasal**		**Temporal**	
	***F***	***p*-Value**	***F***	***p*-Value**	***F***	***p*-Value**	***F***	***p*-Value**	***F***	***p*-Value**
**SUPERFICIAL RETINAL VD**										
Signal strength	5.139	0.028^*^	0.645	0.426	0.434	0.651	2.686	0.108	1.059	0.309
Groups	2.109	0.133	0.671	0.516	0.124	0.726	0.954	0.393	5.050	0.011^*^
Inactive CMVR vs. controls		0.148		0.759		1.000		0.541		0.028^*^
Active CMVR vs. controls		1.000		1.000		1.000		1.000		0.115
Active VS Inactive CMVR		1.000		1.000		1.000		1.000		1.000
**INNER RETINAL VD**										
Signal strength	9.002	0.004^*^	0.520	0.474	6.144	0.017^*^	1.509	0.226	0.442	0.509
Groups	3.990	0.025^*^	0.838	0.439	4.732	0.014^*^	1.040	0.362	5.600	0.007^*^
Inactive CMVR vs. controls		0.030^*^		1.000		0.165		0.495		0.160
Active CMVR vs. controls		0.548		0.644		0.030^*^		1.000		0.012^*^
Active vs. inactive CMVR		1.000		1.000		0.832		1.000		0.520
**CVI**										
Signal strength	1.594	0.213	0.004	0.951	0.135	0.715	2.940	0.093	0.173	0.680
Groups	4.877	0.012^*^	4.398	0.018^*^	5.652	0.007^*^	4.851	0.012^*^	2.152	0.128
Inactive CMVR vs. controls		0.011^*^		0.017^*^		0.005^*^		0.012^*^		0.134
Active CMVR vs. controls		1.000		0.824		1.000		1.000		1.000
Active vs. inactive CMVR		0.166		1.000		0.232		0.141		1.000
**FAZ**										
Signal strength	2.730	0.104								
Groups	3.117	0.053								
Inactive CMVR vs. controls		0.385								
Active CMVR vs. controls		0.856								
Active vs. inactive CMVR		0.053								

*OCTA, optical coherence tomography angiography; FAZ, foveal avascular zone; VD, vessel density; CVI, choroidal vascularity index; RNFL, retinal nerve fiber layer; GCL, ganglion cell layer; IPL, inner plexiform layer; INL, inner nuclear layer; PR, photoreceptor; RPE, retinal pigment epithelium; CMVR, cytomegaloviral retinitis. Statistical analysis was performed using one-way ANOVA and the Bonferroni test*.

* *P-values < 0.05*.

### Macular Structural Changes

Supplementary Table 1 also shows all the structural parameters in both the central fovea and parafoveal areas (superior, inferior, nasal, and temporal) in the three groups. Supplementary Table 2 shows all the statistical results of the structural parameters in those areas. Full choroidal thickness was thinner in some quadrants in the inactive CMVR group. The RNFL thickness and INL thickness were significantly thicker in both the active and inactive CMVR groups in all parafoveal quadrants (all *p* < 0.05). For PR-RPE thickness, no significant differences were found in either the central fovea or parafoveal quadrants between groups. Despite small heterogeneity, decreased choroidal thickness and retinal thickness were shown in the active and inactive CMVR groups.

## Discussion

Unlike CMVR lesions in zone 1, lesions in zone 2 and zone 3 did not cause clinically visible macular necrosis. Our data show that subtle macular structure and microvasculature damage still exist in CMVR eyes without macular-involved necrotic lesions. A shift toward reduced choroidal parameters and thicker retinal thickness was present.

Cytomegalovirus retinitis is an important disease leading to visual impairment and blindness in developing countries and will play a prominent role in the strategy against global blindness (18). Cytomegalovirus retinitis could also occur in immunocompromised patients from other causes, including bone marrow and solid organ transplant recipients, malignancy, primary immunodeficiencies, and ocular corticosteroid injections. Because of similar mechanisms resulting from deprived T lymphocytes, CMVR in HIV- and non-HIV-immunosuppressed patients was found to be similar in most literature (19, 20). For eyes with CMVR, human cytomegalovirus (HCMV) replication coexists with mild smoldering (“persistent”) inflammation; pathological changes involve retinal necrosis of all layers of the retina, manifested as groups of cell swelling, membrane damage, and cellular exudates (21). It is unclear whether these changes affect the macular area without clinically visible CMVR lesions.

This study provided a quantitative assessment of macular retinochoroidal vasculature in eyes with CMVR using SS-OCTA, which was surprisingly limited in previous studies. Patients with CMVR were further divided into active and inactive CMVR because of different retinal findings in different period. The manifestation of active CMVR lesion was retinal edema with opaque (whiteness) borders. After anti-cytomegalovirus treatment, border opacity of active CMVR lesions was decreased to inactive scars (15), but subsequent immune recovery uveitis (IRU) will appear in different degree.

Wongchaisuwat studied macular vascular changes in eyes with CMVR without macular involvement. Their results showed decreased VD in both the superficial and deep retinal capillary plexus (22). We also showed decreased superficial and inner retinal VD in certain quadrants. Although the segmentation definition varies between studies, different results may be mainly attributed to different OCTA devices. For the OCTA device, the scanning rate and laser wavelength were higher in our study: 200,000 vs. 70,000 scans/s, 1,050 vs. 840 nm. Optical coherence tomography angiography signal strength is a quality-image measure. Previous studies described only the cut-off value of the signal strength index (23), qualitative judgement for signal detection (6), or degree of vitreous haze (22). We showed a quantitative judgement of the signal strength. The signal strength in eyes with CMVR, especially inactive CMVR, was significantly weaker than that in the control group. These results also suggested the opacity of dioptric media in eyes with inactive CMVR, which were mainly due to vitritis.

Inflammatory changes, including anterior uveitis, vitritis, papillitis, CME, or epiretinal membrane, could occur in active CMVR (cytomegalovirus-immune recovery retinitis, CMV-IRR) or healed/inactive CMVR (IRU) (24). Kim et al. (6) also showed using SD-OCTA that there was significantly lower density and complexity of capillary perfusion in eyes with uveitis. According to another previous study about intermediate uveitis, the reduced central VD presumably contributes to the presence of CME (25). Four eyes in this study showed CME: three eyes with inactive CMVR and one eye with active CMVR. This may play a role in explaining the changes in this study. Due to our small sample size, we cannot draw firm conclusions regarding subtle macular changes. Large-scale studies aimed at multiple-factor analysis can further improve our understanding of capillary changes.

In addition, SS-OCTA with a 1,050-nm wavelength showed advantages over 840-nm spectral-domain (SD) OCTA in accurate measurements of full-thickness choroid and choroidal structures (26, 27). Three-dimensional CVI with SS-OCTA has contributed greatly to choroidal diseases by volumetrically assessing the choroidal vasculature (17). The choroidal parameters, including CVI and full choroidal thickness in certain quadrants, were decreased in inactive CMVR, showing a sign of choroidal atrophy in resolved infection. Except for PR-RPE thickness, the retinal thickness (full retina, RNFL-GCL-IPL, RNFL, GCL-IPL, INL) in certain quadrants was increased in active or inactive CMVR, showing slight retinal thickening. The structural features in areas of CMVR or macular-involving CMVR have been studied (12, 28, 29). As far as we know, chorioretinal structural changes in the macular area away from CMVR lesions have rarely been studied.

Limitations exist in our study. This is a cross-sectional designed study with a small sample size. There are absences of visual function parameter (best corrected visual acuity or microperimetry for example) and their correlation with OCT and OCTA findings. Further long-term studies assessing longitudinal alterations in the macular structure and microvasculature and large-scale studies aimed at multiple-factor analysis can further improve our understanding of capillary changes in CMVR. Moreover, although the OCTA results in this study were conducted by an experienced retina specialist, there might be a bias between the different operators. With the presence of control group, active and inactive CMVR not involving the macula, our data verified the subtle macular structure and microvascular damages in CMVR eyes without macular-involved necrotic lesions, and offered a foundation for future retinochoroidal vasculature studies and applications for eyes with CMVR using OCTA.

## Conclusions

The OCTA study conducted on changes in the macula and choroid is very valid. Our study showed subtle macular damage, including lower macular chorioretinal vasculature, decreased full choroidal thickness, and thicker retinal thickness, in CMVR eyes without macular-involved necrotic lesions. Using SS-OCTA with a 1,050-nm wavelength, both macular structure and microvasculature can be monitored accurately and may aid in the prognosis of CMVR.

## Data Availability Statement

The original contributions presented in the study are included in the article/Supplementary Material, further inquiries can be directed to the corresponding author/s.

## Ethics Statement

The studies involving human participants were reviewed and approved by the ethics committee of Beijing Youan Hospital, Capital Medical University. The patients/participants provided their written informed consent to participate in this study.

## Author Contributions

K-FD and W-BW contributed to the conception of the protocol. K-FD and CC obtained the dataset. K-FD, L-YX, and W-JK analyzed the data. K-FD and X-JH contributed to writing the first draft of the manuscript and to the revision and editing of the present version of the manuscript. X-JH and W-BW commented on the manuscript and gave final approval of the version to be published. All authors contributed to the article and approved the submitted version.

## Conflict of Interest

The authors declare that the research was conducted in the absence of any commercial or financial relationships that could be construed as a potential conflict of interest.

## Publisher's Note

All claims expressed in this article are solely those of the authors and do not necessarily represent those of their affiliated organizations, or those of the publisher, the editors and the reviewers. Any product that may be evaluated in this article, or claim that may be made by its manufacturer, is not guaranteed or endorsed by the publisher.
