# Supplementary material for: Macular Structure and Microvasculature Changes in AIDS-Related Cytomegalovirus Retinitis Using Optical Coherence Tomography Angiography
Source: Front Med (Lausanne). 2021 Aug 13;8:696447. doi: 10.3389/fmed.2021.696447 (PMC8407073; doi:10.3389/fmed.2021.696447)
Supplement: Supplementary file 1 [file Table_1.DOCX]

**Supplementary table 1**. Summary of microvascular and structural macular OCTA parameters

|  | AIDS patients with active CMVR (mm^2^) | AIDS patients with inactive CMVR | Controls |
| --- | --- | --- | --- |
|  |  |  |  |
| Signal Strength |  |  |  |
| 3×3 mm scan pattern | 142.84 ± 72.38 | 82.88 ± 56.87 | 263.33 ± 105.71 |
| 6×6 mm scan pattern | 218.33 ± 155.17 | 96.30 ± 71.49 | 243.63 ± 105.46 |
| FAZ | 0.44 ± 0.24 | 0.32 ± 0.11 | 0.34 ± 0.11 |
| Superficial retinal VD |  |  |  |
| Central fovea | 30.76 ± 6.80 | 30.72 ± 12.85 | 27.66 ± 9.52 |
| Superior parafovea | 77.59 ± 3.79 | 75.20 ± 6.54 | 78.49 ± 4.78 |
| Inferior parafovea | 75.03 ± 6.08 | 72.21 ± 7.42 | 78.52 ± 4.45 |
| Nasal parafovea | 76.12 ± 3.29 | 71.61 ± 7.95 | 76.52 ± 4.70 |
| Temporal parafovea | 68.33 ± 4.83 | 67.73 ± 6.98 | 74.54 ± 4.56 |
| Inner retinal VD |  |  |  |
| Central fovea | 39.63 ± 9.78 | 37.66 ± 12.76 | 33.46 ± 12.76 |
| Superior parafovea | 81.77 ± 3.53 | 82.92 ± 4.69 | 84.18 ± 2.83 |
| Inferior parafovea | 78.84 ± 6.58 | 79.57 ± 4.76 | 83.86 ± 2.25 |
| Nasal parafovea | 81.80 ± 1.63 | 78.43 ± 7.00 | 81.93 ± 2.90 |
| Temporal parafovea | 74.65 ± 6.61 | 77.46 ± 5.22 | 81.10 ± 2.86 |
| CVI |  |  |  |
| Central fovea | 0.49 ± 0.09 | 0.35 ± 0.14 | 0.49 ± 0.07 |
| Superior parafovea | 0.41 ± 0.07 | 0.35 ± 0.16 | 0.48 ± 0.09 |
| Inferior parafovea | 0.48 ± 0.05 | 0.35 ± 0.18 | 0.51 ± 0.08 |
| Nasal parafovea | 0.51 ± 0.02 | 0.34 ± 0.17 | 0.51 ± 0.08 |
| Temporal parafovea | 0.43 ± 0.03 | 0.37 ± 0.14 | 0.46 ± 0.08 |
| Retinal thickness |  |  |  |
| Central fovea | 320.75 ± 36.96 | 337.15 ± 112.89 | 262.69 ± 17.62 |
| Superior parafovea | 406.75 ± 23.5 | 382.38 ± 53.70 | 349.00 ± 17.34 |
| Inferior parafovea | 390.25 ± 22.10 | 375.92 ± 64.33 | 342.91 ± 18.07 |
| Nasal parafovea | 389.75 ± 7.67 | 389 ± 80.78 | 346.59 ± 17.14 |
| Temporal parafovea | 378 ± 18.09 | 367.69 ± 66.46 | 333.06 ± 16.50 |
| Choroidal thickness |  |  |  |
| Central fovea | 400.5 ± 41.32 | 292.46 ± 121.47 | 410.84 ± 93.93 |
| Superior parafovea | 382.5 ± 32.43 | 297.92 ± 117.52 | 399.72 ± 104.03 |
| Inferior parafovea | 400.75 ± 37.20 | 291.08 ± 118.16 | 403.59 ± 108.70 |
| Nasal parafovea | 267 ± 157.29 | 258 ± 116.17 | 382.88 ± 99.52 |
| Temporal parafovea | 405.25 ± 33.87 | 301.69 ± 113.30 | 401.97 ± 89.14 |
| RNFL-GCL-IPL |  |  |  |
| Central fovea | 59.25 ± 14.77 | 51.92 ± 8.59 | 43 ± 10.31 |
| Superior parafovea | 157.75 ± 9.81 | 132.62 ± 30.31 | 126.06 ± 10.68 |
| Inferior parafovea | 147 ± 6.06 | 132.15 ± 28.77 | 125.03 ± 10.83 |
| Nasal parafovea | 144 ± 7.48 | 129.85 ± 27.62 | 119.69 ± 10.12 |
| Temporal parafovea | 120.5 ± 14.34 | 116.77 ± 25.45 | 112.84 ± 8.62 |
| RNFL |  |  |  |
| Central fovea | 16.25 ± 1.26 | 15.62 ± 2.93 | 14.34 ± 0.90 |
| Superior parafovea | 52.75 ± 7.97 | 38.69 ± 9.39 | 30.38 ± 5.98 |
| Inferior parafovea | 45 ± 2.94 | 38.92 ± 7.81 | 30.66 ± 3.05 |
| Nasal parafovea | 37.75 ± 7.27 | 34.69 ± 7.81 | 25.34 ± 2.16 |
| Temporal parafovea | 26.25 ± 2.22 | 25 ± 3.27 | 20.47 ± 1.48 |
| GCL-IPL |  |  |  |
| Central fovea | 42.75 ± 13.96 | 36.23 ± 10.35 | 28.78 ± 10.03 |
| Superior parafovea | 104.75 ± 5.12 | 94.08 ± 22.70 | 94.81 ± 8.33 |
| Inferior parafovea | 102 ± 4.08 | 93.23 ± 23.96 | 94.28 ± 8.24 |
| Nasal parafovea | 106.5 ± 5.26 | 95.15 ± 22.88 | 94.19 ± 8.49 |
| Temporal parafovea | 94.25 ± 12.20 | 91.69 ± 24.36 | 92.28 ± 7.83 |
| INL |  |  |  |
| Central fovea | 38 ± 9.09 | 36.62 ± 18.88 | 27.97 ± 35.99 |
| Superior parafovea | 64.75 ± 4.57 | 60 ± 15.18 | 44.58 ± 3.30 |
| Inferior parafovea | 59.75 ± 5.19 | 59.15 ± 19.45 | 44.29 ± 4.14 |
| Nasal parafovea | 60.25 ± 4.19 | 57.85 ± 13.17 | 43.81 ± 3.91 |
| Temporal parafovea | 55 ± 1.41 | 56.15 ± 10.76 | 42.55 ± 3.62 |
| PR-RPE |  |  |  |
| Central fovea | 223.5 ± 17.31 | 249.54 ± 108.61 | 198.22 ± 8.419 |
| Superior parafovea | 184 ± 13.17 | 182.77 ± 36.73 | 181.41 ± 23.21 |
| Inferior parafovea | 183.5 ± 13.53 | 184.08 ± 28.76 | 173.34 ± 10.52 |
| Nasal parafovea | 185.5 ± 7 | 200 ± 51.91 | 182.81 ± 11.45 |
| Temporal parafovea | 202 ± 28.91 | 195.54 ± 47.78 | 180.72 ± 19.33 |

*OCTA: optical coherence tomography angiography; FAZ: foveal avascular zone; VD: vessel density; CVI: choroidal vascularity index; RNFL: retinal nerve fiber layer; GCL: ganglion cell layer; IPL: inner plexiform layer; INL: inner nuclear layer; PR: photoreceptor; RPE: retinal pigment epithelium; CMVR: cytomegaloviral retinitis.*
