# Supplementary material for: Macular Structure and Microvasculature Changes in AIDS-Related Cytomegalovirus Retinitis Using Optical Coherence Tomography Angiography
Source: Front Med (Lausanne). 2021 Aug 13;8:696447. doi: 10.3389/fmed.2021.696447 (PMC8407073; doi:10.3389/fmed.2021.696447)
Supplement: Supplementary file 2 [file Table_2.DOCX]

**Supplementary table 2**. Summary of the statistical results of OCTA parameters about macular structure

| Retinal thickness | Central Fovea | | Superior | | Inferior | | Nasal | | Temporal | |
| --- | --- | --- | --- | --- | --- | --- | --- | --- | --- | --- |
|  | F | p-value | F | p-value | F | p-value | F | p-value | F | p-value |
| Signal strength | 0.023 | 0.880 | 0.007 | 0.936 | 0.076 | 0.783 | 0.089 | 0.767 | 0.107 | 0.745 |
| Groups | 5.892 | 0.005^*^ | 0.080 | 0.923 | 1.017 | 0.370 | 4.400 | 0.018^*^ | 4.970 | 0.011^*^ |
| Inactive CMVR VS Controls |  | 0.007^*^ |  | 1.000 |  | 0.484 |  | 0.036^*^ |  | 0.042^*^ |
| Active CMVR VS Controls |  | 0.233 |  | 1.000 |  | 1.000 |  | 0.207 |  | 0.079 |
| Active VS Inactive CMVR |  | 1.000 |  | 1.000 |  | 1.000 |  | 1.000 |  | 1.000 |
| Choroidal thickness | Central Fovea | | Superior | | Inferior | | Nasal | | Temporal | |
|  | F | p-value | F | p-value | F | p-value | F | p-value | F | p-value |
| Signal strength | 0.944 | 0.336 | 1.601 | 0.212 | 0.000 | 0.999 | 0.037 | 0.848 | 0.232 | 0.632 |
| Groups | 3.198 | 0.050 | 1.643 | 0.205 | 3.548 | 0.037^*^ | 4.997 | 0.011 | 3.154 | 0.052 |
| Inactive CMVR VS Controls |  | 0.048^*^ |  | 0.231 |  | 0.035^*^ |  | 0.023 |  | 0.055 |
| Active CMVR VS Controls |  | 1.000 |  | 1.000 |  | 1.000 |  | 0.164 |  | 1.000 |
| Active VS Inactive CMVR |  | 0.402 |  | 0.989 |  | 0.302 |  | 1.000 |  | 0.294 |
| RNFL-GCL-IPL | Central Fovea | | Superior | | Inferior | | Nasal | | Temporal | |
|  | F | p-value | F | p-value | F | p-value | F | p-value | F | p-value |
| Signal strength | 6.667 | 0.013^*^ | 1.804 | 0.186 | 0.570 | 0.454 | 1.393 | 0.244 | 0.251 | 0.619 |
| Groups | 10.652 | < 0.001^*^ | 6.458 | 0.003^*^ | 3.493 | 0.039^*^ | 5.581 | 0.007^*^ | 0.757 | 0.475 |
| Inactive CMVR VS Controls |  | 0.001^*^ |  | 0.306 |  | 0.454 |  | 0.094 |  | 1.000 |
| Active CMVR VS Controls |  | 0.005^*^ |  | 0.004^*^ |  | 0.056 |  | 0.019^*^ |  | 1.000 |
| Active VS Inactive CMVR |  | 1.000 |  | 0.168 |  | 0.698 |  | 0.840 |  | 1.000 |
| RNFL | Central Fovea | | Superior | | Inferior | | Nasal | | Temporal | |
|  | F | p-value | F | p-value | F | p-value | F | p-value | F | p-value |
| Signal strength | 0.464 | 0.158 | 4.830 | 0.033^*^ | 1.312 | 0.258 | 3.147 | 0.083 | 0.798 | 0.377 |
| Groups | 3.754 | 0.031^*^ | 24.643 | < 0.001^*^ | 24.786 | <0.001^*^ | 26.960 | <0.001^*^ | 25.683 | <0.001^*^ |
| Inactive CMVR VS Controls |  | 0.122 |  | <0.001^*^ |  | <0.001^*^ |  | <0.001^*^ |  | <0.001^*^ |
| Active CMVR VS Controls |  | 0.120 |  | <0.001^*^ |  | <0.001^*^ |  | <0.001^*^ |  | <0.001^*^ |
| Active VS Inactive CMVR |  | 1.000 |  | 0.025^*^ |  | 0.236 |  | 1.000 |  | 1.000 |
| GCL-IPL | Central Fovea | | Superior | | Inferior | | Nasal | | Temporal | |
|  | F | p-value | F | p-value | F | p-value | F | p-value | F | p-value |
| Signal strength | 5.990 | 0.018^*^ | 1.002 | 0.322 | 0.258 | 0.614 | 0.612 | 0.438 | 0.169 | 0.683 |
| Groups | 7.838 | 0.001^*^ | 1.062 | 0.354 | 0.571 | 0.569 | 1.556 | 0.222 | 0.043 | 0.958 |
| Inactive CMVR VS Controls |  | 0.007^*^ |  | 1.000 |  | 1.000 |  | 1.000 |  | 1.000 |
| Active CMVR VS Controls |  | 0.021^*^ |  | 0.461 |  | 0.881 |  | 0.266 |  | 1.000 |
| Active VS Inactive CMVR |  | 1.000 |  | 0.934 |  | 1.000 |  | 0.768 |  | 1.000 |
| INL | Central Fovea | | Superior | | Inferior | | Nasal | | Temporal | |
|  | F | p-value | F | p-value | F | p-value | F | p-value | F | p-value |
| Signal strength | 1.334 | 0.254 | 0.108 | 0.744 | 0.153 | 0.697 | 0.003 | 0.959 | 0.243 | 0.625 |
| Groups | 0.992 | 0.379 | 16.641 | <0.001^*^ | 7.560 | 0.002^*^ | 16.010 | <0.001^*^ | 19.821 | <0.001^*^ |
| Inactive CMVR VS Controls |  | 0.564 |  | <0.001^*^ |  | 0.006^*^ |  | <0.001^*^ |  | <0.001^*^ |
| Active CMVR VS Controls |  | 1.000 |  | <0.001^*^ |  | 0.032^*^ |  | 0.001^*^ |  | 0.002^*^ |
| Active VS Inactive CMVR |  | 1.000 |  | 0.919 |  | 1.000 |  | 1.000 |  | 1.000 |
| PR-RPE | Central Fovea | | Superior | | Inferior | | Nasal | | Temporal | |
|  | F | p-value | F | p-value | F | p-value | F | p-value | F | p-value |
| Signal strength | 0.238 | 0.628 | 0.166 | 0.685 | 0.051 | 0.822 | 0.021 | 0.885 | 0.550 | 0.462 |
| Groups | 2.196 | 0.123 | 0.072 | 0.931 | 1.736 | 0.188 | 1.072 | 0.351 | 1.962 | 0.152 |
| Inactive CMVR VS Controls |  | 0.138 |  | 1.000 |  | 0.299 |  | 0.453 |  | 0.314 |
| Active CMVR VS Controls |  | 1.000 |  | 1.000 |  | 0.827 |  | 1.000 |  | 0.526 |
| Active VS Inactive CMVR |  | 1.000 |  | 1.000 |  | 1.000 |  | 1.000 |  | 1.000 |

*OCTA: optical coherence tomography angiography; VD: vessel density; CVI: choroidal vascularity index; RNFL: retinal nerve fiber layer; GCL: ganglion cell layer; IPL: inner plexiform layer; INL: inner nuclear layer; PR: photoreceptor; RPE: retinal pigment epithelium; CMVR: cytomegaloviral retinitis. Statistical analysis was performed using One-Way ANOVA and Bonferroni test. P values < 0.05 are indicated by asterisk (*).*
